# Diagnosis and Management of Intraoperative Colorectal Anastomotic Leaks: A Global Retrospective Patient Chart Review Study

**DOI:** 10.1155/2017/3852731

**Published:** 2017-06-14

**Authors:** A. Schiff, S. Roy, M. Pignot, S. K. Ghosh, E. J. Fegelman

**Affiliations:** ^1^Johnson & Johnson (Ethicon), Cincinnati, OH, USA; ^2^Johnson & Johnson (Ethicon), Somerville, NJ, USA; ^3^Kantar Health GmbH, Munich, Germany

## Abstract

**Background:**

This targeted chart review study reports the first ever detailed global account of clinical approaches adopted to detect and manage anastomotic leaks identified during surgery in routine clinical practice.

**Method:**

156 surgeons from eight countries retrospectively extracted data from surgical records of 458 patients who underwent colorectal surgery with an identified intraoperative leak at the circular anastomosis. Demographic details, procedures, and outcomes were analyzed descriptively, by country.

**Results:**

Most surgeries were performed laparoscopically (57.6%), followed by open surgeries (35.8%). The burden of intraoperative leaks on the healthcare system is driven in large part by the additional interventions such as using a sealant, recreating the anastomosis, and diverting the anastomosis to a colostomy bag, undertaken to manage the leak. The mean duration of hospitalization was 19.9 days. Postoperative anastomotic leaks occurred in 62 patients (13.5%), most frequently 4 to 7 days after surgery. Overall, country-specific differences were observed in patient characteristics, surgical procedures, method of diagnosis of intraoperative leak, interventions, and length of hospital stay.

**Conclusion:**

The potential cost of time and material needed to repair intraoperative leaks during surgery is substantial and often hidden to the healthcare system, potentially leading to an underestimation of the impact of this complication.

## 1. Introduction

Colorectal resection surgery is often performed to remove malignant colon tissue in patients with colon cancer or rectal cancer. The healthy sections of the colon are reconnected by an anastomosis, which can be created by suturing or by using a stapling device, and surgery can be performed using an open or a minimally invasive approach. A number of linear and circular staplers are currently on the market to facilitate a laparoscopic procedure. Both suturing and stapling have advantages and disadvantages [1, 2]. Recent results suggest that a combination of stapling and hand-sewn reinforcement of the staple line may be useful [3]. Furthermore, the evidence suggests that laparoscopic surgery may be advantageous compared to open surgery [4–10]. In particular, postoperative anastomotic leaks have been found to occur less frequently after laparoscopic surgery than after open surgery [11, 12]. However, these findings remain controversial, as other studies did not observe a significant advantage of laparoscopic versus open surgery or of stapled versus sutured anastomoses [13–18].

Anastomotic leaks are one of the most severe complications of colorectal resections. Postoperative anastomotic leaks have serious sequelae like infection, abscesses, or peritonitis and can be difficult to detect [19]. They have been shown to increase the patient's risk of cancer recurrence and death [20–23].

Intraoperative leak testing is often performed to assess the integrity of the anastomosis. A recent systematic review found that intraoperative testing was performed in 86.5% of patients in the reviewed studies and intraoperative leaks were identified in 6.3% of all patients who were tested [24]. A positive leak test requires immediate intervention, which may consist of one or a combination of several procedures, for example, suturing, the use of surgical sealant, or recreation of the anastomosis. Intraoperative anastomotic leaks are usually detected by insufflation of air (air leak test) or by instillation of methylene blue-stained saline (dye test). As most current literature predominantly focuses on postoperative leaks, further evaluations of the rates, circumstances, and the management of intraoperative anastomotic leaks are warranted.

The present study was a retrospective, multicentre patient chart review study. The aim was to describe the methods of identification, treatment, and management of intraoperative anastomotic leaks during colorectal resection surgery performed with a circular stapler. A secondary goal was to assess the incidence of postoperative leaks following the detection and management of an intraoperative leak. The study documented surgical practice in eight countries to assess how surgeons identify and manage intraoperative anastomotic leaks.

## 2. Methods

This study followed Good Epidemiological Practice (GEP) in describing the identification of intraoperative anastomotic leaks and the treatment and overall management of patients with such a leak during colorectal resection surgery performed with a circular stapler.

The study was performed in eight countries [(United States (USA), France, Germany, Italy, United Kingdom (UK), China, Japan, and South Korea)]. Surgeons were recruited from a Lightspeed All Global (AG) panel, based on defined criteria including their specialty and operating record, to ensure a certain level of surgical experience in the participating physicians. AG panels include telephone-recruited physicians for online research who explicitly stated their willingness to contribute to research studies by providing opinions and access to treatment data on a regular basis. Validation procedures and background checks were performed to ensure eligibility of the respondents to participate in this study. The survey was conducted in a double-blinded manner, where surgeons were not aware of who the study sponsors were, and the sponsor was unaware of the identity of the surgeons beyond their specialty, country, and length of surgical practice.

Data were collected from surgical records of patients (≥18 years) if they had a positive anastomotic leak test during a planned colorectal resection procedure (left hemicolectomy, sigmoidectomy, or anterior rectum resection) where anastomosis was established with a circular stapler. Patients were excluded if they underwent a planned end/loop colostomy or loop ileostomy, if another major procedure (e.g., liver resection, hysterectomy, and bladder resection) was performed along with the colorectal resection, or if they had been diagnosed with Crohn's Disease or Inflammatory Bowel Disease, thus focusing primarily on patients with diagnoses of colon or rectal cancer.

In line with the objectives of the study, data on several demographic, epidemiological, and technical parameters related to the management of an intraoperative anastomotic leak were collected using an electronic case report form (eCRF). These parameters included information about surgeons (including specialty, experience) and patients (including demographics, disease characteristics, prior therapies, medications, and comorbidities), technical details of surgical procedures, occurrence, and management of anastomotic leaks (intraoperative and postoperative, including time taken to manage leak, interventions), patient survival, and details of hospitalisations. Inclusion and exclusion criteria, as well as feasibility checks, were implemented in the electronic data capture system (EDC) for data collection.

The sampling procedure of this study was designed to generate a qualitative assessment of treatment patterns and not necessarily to achieve a representative sample from a basic population for testing any hypotheses. To reduce effects of practice patterns of specific centres, >10 centres per country were included and around 3 patients per centre planned to be documented. All data were stratified by country to detect country-specific variability. As this was not a controlled study, the sample size was defined according to practical feasibility considerations.

The statistical analyses were primarily descriptive. Where statistical tests (Chi-square tests) were performed for selected variables, *p* values are provided. No imputation of missing values was performed. Percentages were calculated as a proportion of each category in its entirety, including missing values. All documented patients were included in the analysis set. Data are generally presented as mean ± standard deviation (SD) and further details (median, range) are provided in Supplemental Tables in Supplementary Material available online at https://doi.org/10.1155/2017/3852731.

## 3. Results

### 3.1. Participating Surgeons

A total of 156 surgeons participated in this study: 11 from the USA, 15 from France, 13 from Germany, 17 from Italy, 33 from the UK, 18 from China, 37 from Japan, and 12 from South Korea. Most study centres were represented by one surgeon from each centre. The mean (±SD) experience of the surgeons in colorectal surgeries was 16.3 ± 7.3 years, with a broad range from a minimum of 3 years to a maximum of 31 years overall. At the country level, the mean experience ranged from 10.3 ± 7.0 years in China to 23.1 ± 3.0 years in Japan. The most common medical specialties were general surgeon (35.3% of all surgeons), colorectal surgeon (28.2%), and gastroenterologist performing colorectal surgery (25.0%).

The participating surgeons were asked a series of questions about their experience with colorectal surgeries. The surgeons reported performing on average 18.7 ± 20.6 colorectal surgeries per month. In addition, they reported having performed 65.4 ± 51.4 colorectal resections using a circular stapler to create an anastomosis in the 12 months prior to the survey.

### 3.2. Surgical Records

Surgical records of 458 patients were included in the study. The number of patients was comparable across the participating countries, ranging from 52 patients included in South Korea to 64 in the UK. Each surgeon reported on a mean number of 3.0 ± 3.1 patients. Surgeons in the USA reported on the highest mean number of patients (4.9 ± 4.2), while the lowest number was contributed per surgeon in Japan (1.5 ± 1.1). Most frequently, the surgeons documented only 1 patient each (83 surgeons, 53.2%), followed by 23 surgeons (14.7%) reporting 3 patients each and 21 surgeons (13.5%) reporting 10 patients each.

### 3.3. Patient Demographic and Disease Characteristics

The patient demographic and disease characteristics are presented overall in Table 1 and by country in Supplemental Tables 1 and 2. Overall, the patients' mean age was 64.5 ± 9.7 years at the time of surgery. The youngest patient was 18 years, the oldest was 88 years old. Most patients were in the ≥60 to <70 years (179 patients, 39.1%) and the ≥70 to <80 years (124 patients, 27.1%) age groups. The mean age of patients getting colorectal surgery was highest in Germany (66.6 ± 8.4 years) and the UK (66.4 ± 8.9 years) and lowest in China (62.1 ± 9.5 years) and Italy (62.9 ± 14.8 years). The majority of patients were male, overall (63.5%) and in each country (range from 51.8% in France to 75.0% in Japan). Most patients were White (56.6%) or Asian (36.9%). The overall mean body mass index (BMI) was 25.4 ± 4.4 kg/m^2^. The highest mean BMIs were observed in the USA (27.9 ± 6.1 kg/m^2^) and the UK (27.9 ± 4.9 kg/m^2^) and the lowest in South Korea (22.0 ± 3.1 kg/m^2^) and Japan (23.7 ± 2.4 kg/m^2^). Overall, 50.9% of patients with reports on smoking staus had never smoked and 33.7% had permanently stopped smoking before surgery. A further 15.4% were smokers at the time of surgery.

Colon cancer was the most frequently reported primary type of cancer (65.5% of patients), followed by rectal cancer (34.1%). When evaluating the type of cancer by country, the most notable differences to the overall results were seen in France and in the USA, where the prevalence of colon cancer in their respective samples was higher (91.1% and 87.0% of patients, resp.), and in South Korea and China, where rectal cancer was more common (50.9% and 51.9% of patients, resp.) than in the other countries.

The pathological stage of the patients' cancer before resection was documented according to the TNM (Tumour Node Metastasis) classification system of malignant tumours. Overall, the most frequently documented TNM stages were T3 (31.7% of all patients) and T2a (24.7%). Node classifications were most commonly N0 (44.1%) and N1 (37.6%) and metastasis classifications were mostly M0 (89.7%) (Table 1). The TNM stages tended to be lower (better) in France and the UK and higher (worse) in China and South Korea (Supplemental Table 2).

Neoadjuvant chemotherapy prior to colorectal surgery was received by 67 of 458 patients (14.6%) overall. This was most common in the USA (19 of 54 patients, 35.2%) and Italy (15 of 57 patients, 26.3%) and least common in the UK (4 of 64 patients, 6.3%) and Japan (4 patients, 7.1%). Furthermore, 56 of 458 patients (12.2%) underwent radiation therapy prior to resection. This was most frequently documented in Italy (19 patients, 33.3%) and in the USA (12 patients, 22.2%). Only 1 patient in Japan (1.8%) and no patient in China received radiation treatment. The most frequently documented concomitant medications were antibiotics (275 of 458 patients, 60.0%). By country, notably higher antibiotics usage was seen in the USA (92.6% of patients) and in Japan (78.6%) and lower antibiotics usage in South Korea (11.5%).

### 3.4. Colorectal Resection Procedures

**Figure 1:**
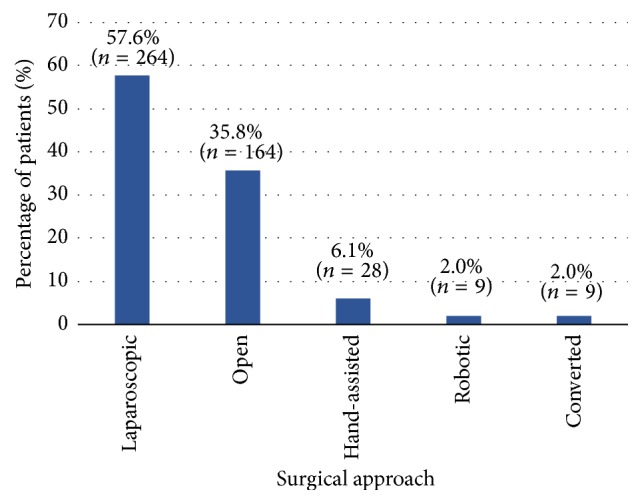
*Surgical approach to colorectal resection procedure (overall, N* = 458). Percentages and absolute numbers of patients with respective approach. Multiple responses were possible (>1 approach documented for 16 patients). Specification of converted approach: 7 patients converted from laparoscopic to open, 1 from hand-assisted to open, 1 from robotic to open. Further details and by-country data provided in Supplemental Table 3. *N*: total number of patients; *n*: number of patients observed per category.

Among the procedures the 458 patients included in this study undergone, anterior rectum resection (131 patients, 28.6%) and laparoscopic left hemicolectomy (127 patients, 27.7%) were most common, followed by laparoscopic sigmoidectomy (107 patients, 23.4%). Open/other left hemicolectomies (47 patients, 10.3%) and open/other sigmoidectomies (46 patients, 10.0%) were performed least often. The surgical approach used during the colorectal resection was mostly laparoscopic (57.6% of all patients), followed by open (35.8%). Hand-assisted (6.1%) and robotic (2.0%) approaches were employed less frequently, and conversions from minimally invasive to open procedures were less frequent (2.0%) as well (Figure 1). Only 1 approach was used in 96.5% of cases (442 patients); only a small number of patients (3.5%) had resections with 2 different surgical approaches. A comparison of laparoscopic versus open approach is explored in more detail in a subgroup analysis presented later. When analysed by country, laparoscopic surgery was performed most frequently in France (78.6% of patients) and least often in the UK (42.2%). Conversely, open surgery was performed most often in the UK (50.0%) and least often in France (14.3%) (Supplemental Table 3).

**Figure 2:**
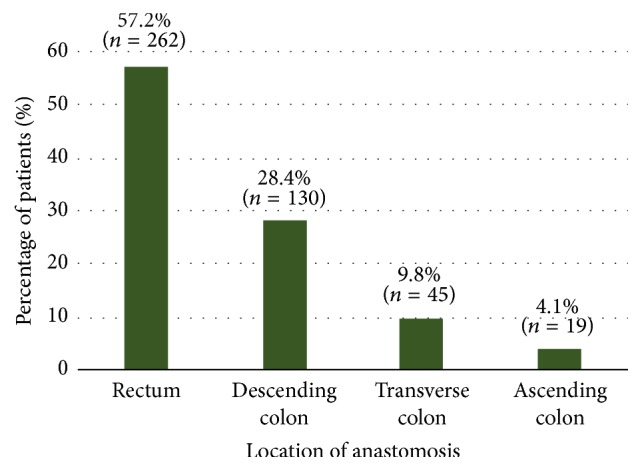
*Location of anastomosis created during colorectal resection procedure (overall, N* = 458). Percentages and absolute numbers of patients with anastomosis in respective location. Further details and by-country data provided in Supplemental Table 4. “Other” locations documented for 2 further patients (both specified by investigator as sigmoid colon). *N*: total number of patients; *n*: number of patients observed per category.

Following the transections, the anastomosis was created. Most frequently, it was located in the rectum (57.2% of all patients), followed by the descending colon (28.4%), the transverse colon (9.8%), and the ascending colon (4.1%) (Figure 2). This distribution was comparable among the countries and reflects the patients' type of cancer. The most notable differences were seen in the USA, with fewer rectal anastomoses (12 of 54 patients, 22.2%) and higher percentages of anastomoses in the descending colon (22 patients, 40.7%), transverse colon (12 patients, 22.2%), and ascending colon (7 patients, 13.0%), compared to the overall results. In Germany, the percentage of anastomoses in the transverse colon was similarly high (13 of 62 patients, 21.0%). In South Korea, the percentage of anastomoses in the rectum was higher than in all other countries (44 of 52 patients, 84.6%), with correspondingly fewer anastomoses in the other locations of the colon (Supplemental Table 4). A comparison of rectal location versus colon locations is explored in more detail in a subgroup analysis presented further below.

The surgeons were asked to document additional details of the procedures performed during creation of the anastomosis: the anvil of the circular stapler was placed intracorporeally in 243 of 458 patients (53.1%) and extracorporeally in 215 patients (46.9%). The lumen on the anvil side was most frequently closed by purse string suture (383 patients, 83.6%) and less frequently by endocutter (74 patients, 16.2%). The circular stapler was usually introduced through the anus (323 patients, 70.5%) and less frequently through a skin incision (132 patients, 28.8%). The spike of the stapler was most frequently introduced through the staple line (248 patients, 54.1%), followed by introduction adjacent to the staple line (182 patients, 39.7%) and through the side for an end-to-end anastomosis (28 patients, 6.1%). Mostly no prefiring interventions were performed at the staple line (395 patients, 86.2%). A buttress was used in 61 patients (13.3%). Anvil and stapler engaged and fired in 450 patients (98.3%). The stapler was most frequently fired by the attending physician (225 patients, 49.1%) or the first assistant (205 patients, 44.8%). Residents or other staff rarely fired the device. For the majority of patients (298 patients, 65.1%), no adjunct interventions were required at the staple line. Oversewing was performed for 139 patients (30.4%) and haemostatic agent/sealant was used in 25 patients (5.5%). A visual confirmation of two complete donuts for the anastomosis was performed for almost all patients (441 patients, 96.3%).

The entire colorectal surgery (from first skin incision to skin closure) took a mean time of 190.1 ± 83.1 minutes overall. This included any time for management of the intraoperative anastomotic leak. By country, the mean total operating time was around 180 to 200 minutes in most countries and was only notably shorter in Germany (156.7 ± 56.7 minutes) and longer in South Korea (237.2 ± 72.4 minutes).

### 3.5. Intraoperative Anastomotic Leaks

All 458 patients included in this study experienced an intraoperative anastomotic leak, as per inclusion criteria. Details of the procedures performed to detect and manage the leak are provided in Table 2 and in Supplemental Table 5. The leak was most frequently diagnosed by a positive air leak test (75.8% of patients). A methylene blue dye test was used less often (22.5%). Notable variability was seen across the countries. Air leak tests were used for all patients in the UK and 92.3% of patients in South Korea. In contrast, dye tests were used more frequently in France (46.4% of patients) and in Italy (45.6%) than in the other countries.

The mean time it took to manage and stop the leak was 21.2 ± 16.8 minutes overall. This time ranged from 12.3 ± 8.2 minutes in Japan and 13.2 ± 13.1 minutes in France to 28.9 ± 19.8 minutes in South Korea and 27.7 ± 20.5 minutes in China. Following the positive test, oversewing of the staple line was the most commonly performed intervention (77.5% of all patients), followed by the use of sealant (17.5%). Ileostomy/colostomy (10.3%) and creation of a new anastomosis (9.4%) were less frequently chosen. The largest differences by country, compared to the overall data, were seen in Germany, where oversewing of the staple line was only performed for 53.2% of patients, while a new anastomosis was created for 24.2% of patients. Sealant was used more frequently in France (33.9% of patients) and Italy (33.3%) than in the other countries. In those cases where the staple line had to be oversewn, a mean number of 3.9 ± 3.2 suture strands were used. For the interventions using sealant, a mean number of 2.2 ± 1.0 sealant tubes were required per patient. A new anastomosis was created by circular stapler in 76.7% of all 43 patients who had this intervention.

In the cases where all the above interventions were used together, the mean required time was reported as 22.0 ± 21.0 minutes per patient. This time was slightly lower for oversewing of the staple line (15.8 ± 10.2 minutes) and for interventions using sealant (14.9 ± 13.1 minutes), while it was notably longer for the creation of a new anastomosis (37.0 ± 21.8 minutes). Ileostomy/colostomy interventions were reported as taking 29.4 ± 23.6 minutes per patient.

Almost all patients survived the colorectal surgery including intraoperative anastomosis leakage treatment (453 of 458 patients, 98.9%). For the 5 patients who died, the mean duration from surgery to death was 13.0 ± 8.2 days.

### 3.6. Postoperative Anastomotic Leaks

**Figure 3:**
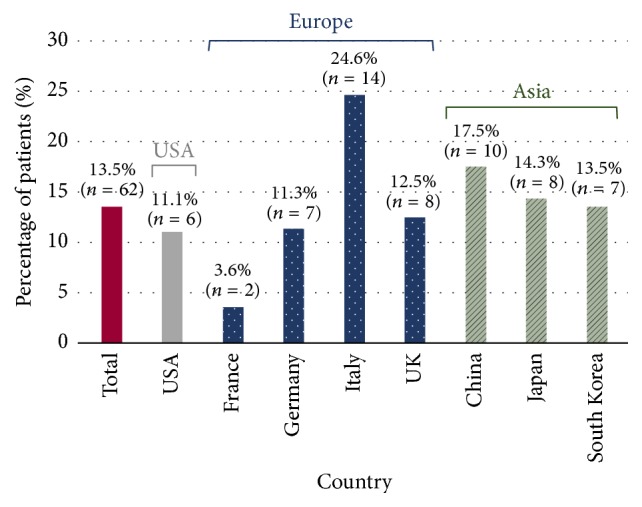
*Occurrence of postoperative anastomotic leaks, overall (N* = 458*) and by country*. Percentages and absolute numbers of patients with postoperative anastomotic leaks. Percentage for each country based on number of patients per country. Further details provided in Supplemental Table 6. *N*: total number of patients; *n*: number of patients observed per category.

Postoperative anastomotic leaks occurred in 62 of the 458 patients (13.5%) overall. Such leaks occurred least frequently in France (2 of 56 patients, 3.6%) and most frequently in Italy (14 of 57 patients, 24.6%) and in China (10 of 57 patients, 17.5%) (Figure 3 and Supplemental Table 6). The postoperative leaks were most frequently diagnosed by clinical leak (42 of the 62 patients with postoperative leak, 67.7%), followed by radiographic leak (20 patients, 32.3%). Diagnosis by clinical leak predominated in France, Italy, China, Japan, and South Korea, while the majority of patients in Germany and the USA were diagnosed by radiographic leak. The two methods were distributed equally among the patients from the UK. The postoperative leaks most frequently occurred 4 to 7 days after surgery (24 of 62 patients, 38.7%), while they occurred 1 to 3 days after surgery in 21 patients (33.9%), 7 to 10 days after surgery in 14 patients (22.6%), and 10 days (or more) after surgery in 3 patients (4.8%). It was of interest to assess whether the interventions the surgeons used during surgery to manage the intraoperative leak had an impact on the occurrence of postoperative leaks. Here, no notable impact was seen for oversewing or sealant use. However, the percentage of patients for whom the intraoperative leak was repaired by creating a new anastomosis was higher among the patients who experienced a postoperative leak (17.7%) than among those patients who did not have a postoperative leak (8.1%).

### 3.7. Duration of Patients' Hospitalisation

The overall mean duration of hospitalisation was 19.9 ± 29.4 days (median 14 days), based on data from 443 patients. Broad variation was seen when analysing this duration by country, with the shortest duration observed in the USA (9.1 ± 5.9 days; median 7 days; *n* = 52) and the longest durations in Germany (25.6 ± 44.3 days; median 15 days; *n* = 58) and in Japan (25.2 ± 23.3 days; median 18 days; *n* = 52). The mean time from hospital admission to colorectal surgery was 4.9 ± 13.1 days overall (median 2 days; *n* = 458) and the mean time from surgery to hospital discharge was 15.2 ± 26.5 days (median 10 days; *n* = 443). The overall mean duration of hospitalisation was higher among the patients who experienced a postoperative leak (31.0 ± 47.1 days; median 20 days; *n* = 57) than among those patients who did not have a postoperative leak (18.3 ± 25.4 days; median 13 days; *n* = 386).

### 3.8. Analysis of the Impact of Laparoscopic versus Open Surgery Approach on Outcomes

Overall, the colorectal resection procedure was performed laparoscopically in 264 of 458 patients (57.6%) and by open approach in 164 patients (35.8%). The most notable, statistically significant differences observed between these two groups are described in the following and details are provided in Supplemental Table 7.

A significant relationship between the patients' country and the surgical approach was observed (*p* value: 0.0004). The proportion of laparoscopic surgeries per country ranged from 45.8% of all surgeries in the UK to 84.6% of all surgeries in France.

Laparoscopic surgery was performed more often (68.8% of patients) than open surgery (31.2%) in patients with colon cancer, while the reverse was the case in patients with rectal cancer (52.8% open, 47.2% laparoscopic). The relationship between type of cancer and surgical approach was significant (*p* value: <0.0001).

Similarly, a significant relationship was observed between the location of the anastomosis and the surgical approach (*p* value: 0.0159). Laparoscopic surgeries predominated among patients with anastomoses in the ascending colon (14 of 17 patients, 82.4%) and transverse colon (34 of 44 patients, 77.3%). The percentage of laparoscopically created anastomoses was lower in the descending colon (77 of 121 patients, 63.6%) and the rectum (137 of 244 patients, 56.1%).

Postoperative anastomotic leaks were observed more frequently in patients after open surgery (32 of 56 patients with postoperative leaks, 57.1%) than after laparoscopic surgery (24 of 56 patients, 42.9%). This relationship between surgery and postoperative leaks was significant (*p* value: 0.0019). However, conclusions from this finding are limited due to the comparably low number of patients with postoperative leaks.

The mean duration of the patients' hospitalisation was observed to be numerically longer in patients who underwent open surgery (23.0 ± 41.9 days), compared to those who underwent laparoscopic surgery (18.9 ± 20.3 days). This finding was supported by longer durations from surgery to hospital discharge after open surgery (19.2 ± 41.2 days) compared to laparoscopic surgery (13.3 ± 13.2 days). However, the time from hospital admission to surgery was observed to be longer for patients undergoing laparoscopic surgery (5.6 ± 15.7 days) compared to open surgery (4.0 ± 8.9 days).

All 5 patients who died during this study had undergone open surgery. However, the number of patients is too small to allow reliable conclusions.

### 3.9. Analysis of the Impact of the Anastomosis Location (Rectum versus Colon) on Outcomes

The locations of anastomoses were stratified by location in rectum and location in colon (including locations in ascending, transverse, and descending colon). Overall, for 262 of 458 patients (57.21%) the anastomosis was created in the rectum and for 196 patients (42.79%) it was located in the colon. The most notable differences observed between these two groups are described in the following and details are provided in Supplemental Table 8.

A statistically significant relationship was observed between the patients' country and the location of anastomosis (*p* value: <0.0001). The percentage of patients with a rectal anastomosis ranged from 22.2% of all patients in the USA to 84.6% of all patients in South Korea. Patients with rectal anastomoses were slightly older (mean 65.6 ± 9.7 years) than those with anastomoses in the colon (63.1 ± 9.5 years).

Surgery took longer for patients with rectal anastomoses (199.4 ± 74.3 min) than for patients with colon anastomoses (177.6 ± 92.5 min).

The patients' mean duration of hospitalisation was numerically longer in patients with rectal anastomoses (22.3 ± 31.5 days) than in patients with colon anastomoses (16.8 ± 26.0 days). This was also reflected in the mean duration from surgery to hospital discharge (rectum: 17.0 ± 27.6 days; colon: 12.8 ± 24.9 days).

A statistically significant relationship was observed between the location of anastomosis and the occurrence of a postoperative anastomotic leak (*p* value: 0.0002). Such leaks were observed more frequently in patients with rectal anastomoses (49 of 62 patients with postoperative leaks, 79.0%) than in patients with colon anastomoses (13 patients, 21.0%).

## 4. Discussion

In this retrospective study, 156 surgeons documented data from medical records of 458 patients who had colorectal resection surgery and were detected having an intraoperative anastomotic leak. More than 10 surgeons and more than 50 patients were included from each of the 8 participating countries, in order to minimize selection bias. The mean experience of the surgeons in colorectal surgeries was approximately 16 years. As such, the data collected in this study were less likely to be affected by surgeons' inexperience, which has previously been reported as one of the risk factors for complications in colorectal surgeries [25]. In the patients' demographic and baseline characteristics, the largest variations by country were seen with regard to BMI and type of cancer, reflecting potential regional variability in diagnostic and surgical practices.

The surgical approach used during the colorectal resection was mostly laparoscopic (57.6%), followed by open (35.8%). Although regional variations exist, this distribution is different from the general estimate of a majority of colorectal procedures being done with an open approach. It is important to note that the surgeons were not directed to report on any particular approach, and as such, this observation could potentially be reflective of the increasing adoption of laparoscopy. Greater use of the laparoscopic approach in appropriately selected patients may deliver superior patient outcomes, shorter recovery time, lower costs, and fewer risks [4, 7, 10].

In line with the patients' documented types of cancer, the anastomoses in this study were most frequently located in the rectum (57.2%), followed by the descending colon (28.4%), the transverse colon (9.8%), and the ascending colon (4.1%). The entire colorectal surgery took a mean time of 190 minutes overall, including the time for detecting and managing the intraoperative anastomotic leak that occurred in all patients. This is only slightly longer than the operative times between 140 and 190 minutes published for similar procedures irrespective of whether a leak occurred [7, 26], indicating that intraoperative management of anastomotic leaks does not have a major impact on operating time. In contrast, a leak that is not detected during surgery and requires secondary postoperative procedures poses a higher clinical and economic burden [27].

In this study, the intraoperative leak was most frequently diagnosed by a positive air leak test. Notable variability was seen across the countries, with dye tests predominating in France and in Italy. The mean time taken to manage and stop the leak was 21 minutes. Oversewing of the staple line was the most commonly performed intervention (77.5% of patients), followed by the use of sealant (17.5%).

Postoperative anastomotic leaks occurred in 13.5% of patients. The postoperative leaks most frequently occurred 4 to 7 days after surgery (38.7% of patients with postoperative leak), while they occurred 1 to 3 days after surgery in 33.9% of patients and later than 7 days after surgery in 27.4% of patients. This reflects practical experience in that most leaks are detected during the first 10 days after surgery and, thus, close monitoring of patients is required during this period in order to detect complications as early as possible [19, 28]. In this study, the intraoperative creation of a new anastomosis after a leak during surgery appeared to increase the patients' risk of a postoperative leak compared to estimates in the literature [12]. However, these findings have to be interpreted with caution owing to the inclusion criteria of the incidence of intraoperative leaks and the low overall number of subjects with postoperative leaks.

The mean time from surgery to hospital discharge was 15 days and the overall duration of hospitalisation was 20 days. Broad variation was observed by country, with the shortest durations generally found in the USA. The overall length of stay observed across all countries was longer than what has been published in comparable settings irrespective of anastomotic leakage; the literature refers to mean hospitalisation times between 5 and 13 days for the entire stay [7, 8, 26, 29]. This suggests that while operative time was not greatly increased by the management of intraoperative leaks, these leaks may have led to longer recovery times and longer postoperative hospitalisation. This is especially true for those patients who experienced a postoperative leak in addition to their intraoperative leak, in line with published research on increased hospitalisation times and, in turn, increased costs after postoperative leaks [27, 30].

A subgroup analysis was done to assess differences between laparoscopic and open surgical approaches. Whether laparoscopic or open surgery was performed was influenced significantly by the following factors: patients' country (laparoscopic surgery most frequent in France, least frequent in UK), type of cancer (more laparoscopic surgeries in colon than in rectal cancer), and location of the anastomosis (more laparoscopic surgeries in upper sections of colon). These findings are likely due to national surgical practices and to differences in the nature of colorectal cancers. Rectal resections are generally seen as more challenging than colon resections; thus, laparoscopic resections may be attempted less frequently in this area. Furthermore, the occurrence of postoperative anastomotic leaks was also significantly influenced by the surgical approach (more frequent in patients after open than after laparoscopic surgery). This is in line with recent publications which suggest that laparoscopic surgery provides better short-term outcomes and fewer complications than open surgery [5, 6, 9].

A further subgroup analysis was done to evaluate the difference between higher and lower locations of the anastomosis (rectum or colon). A significant relationship was found between the anastomotic location and the patients' country (e.g., colon anastomoses predominating in the USA, rectal anastomoses predominating in South Korea). Again, this likely reflects differences in types of cancer based on the ethnic background of the patient populations and differences in surgical practice. In addition, a significant relationship was also found between the anastomotic location and the occurrence of a postoperative anastomotic leak (more frequent in patients with rectal anastomoses). This is in line with current literature showing that the risk of postoperative leaks increases with decreased proximity of the anastomosis to the anal verge [31–33].

While the present study offers a new level of understanding of both the detection and management of intraoperative anastomotic leaks, there are some limitations to this study that should be noted. This study is not powered to test a specific hypothesis or make any comparison between patients or outcomes. The study reports inputs from a self-selected group of surgeons who were interested in participating in a study of this nature and chose to report from patient records that met certain clinical criteria. The study is also observational in nature and may not take into consideration every variable that could impact surgical management choices.

## 5. Conclusions

To conclude, the present study assessed how colorectal surgeons worldwide treat intraoperative anastomotic leaks. Detection and management of intraoperative leaks did appear to have a material impact on operative time and the length of patients' hospital stay was also longer than is typically observed. The additional days in hospital and the materials used for managing the intraoperative leak increase the cost of colorectal surgeries. Furthermore, anastomotic leaks can increase patients' recovery time and their risk of postoperative complications. In this study, country-specific differences were seen not only in patient characteristics, for example, regarding the type of cancer and baseline disease severity, but also regarding surgical procedures, methods of diagnosis of the anastomotic leak, and interventions and regarding the length of hospital stays. Overall, any intraoperative interventions that reduce the incidence rate of intra- or postoperative leaks may help reduce the clinical burden on the patient as well as the economic burden on the healthcare system.

## Supplementary Material

The supplemental tables provide more granular data, primarily by the each country included in the study. A couple of tables also provide additional details when the data are straitified by the surgical approach, or by the location of the anastomosis.

## Figures and Tables

**Table 1:** Summary of patients' demographic and disease characteristics.

Characteristic	Total
*Age (years)*	*N* = 458
Mean (SD)	64.5 (9.7)

*Gender, n (%)*	*N* = 458
Female	167 (36.5%)
Male	291 (63.5%)

*Race, n (%)*	*N* = 458
White	259 (56.6%)
Black/African American	26 (5.7%)
Asian	169 (36.9%)
Hispanic	4 (0.9%)

*BMI (kg/m* ^*2*^)^1^	*N* = 457
Mean (SD)	25.4 (4.4)

*Type of cancer, n (%)*	
Colon cancer	300 (65.5%)
Rectal cancer	156 (34.1%)
Other^2^	2 (0.4%)

*Smoking status, n (%)* ^3^	*N* = 395
Never smoked^4^	201 (50.9%)
Stopped smoking before surgery^5^	133 (33.7%)
Smoking at time of surgery^6^	61 (15.4%)

*TNM stage of cancer, n (%)* ^7^	
Tumour stage T0	18 (3.9%)
T1a	44 (9.6%)
T1b	34 (7.4%)
T2a	113 (24.7%)
T2b	55 (12.0%)
T3	145 (31.7%)
T4	43 (9.4%)

Node stage N0	202 (44.1%)
N1	172 (37.6%)
N2	66 (14.4%)
N3	11 (2.4%)

Metastasis stage M0	411 (89.7%)
M1	31 (6.8%)

Further details and by-country data provided in Supplemental Tables 1 and 2. ^1^No information available due to missing height measurement for 1 patient (from USA). ^2^“Other” types documented for 2 patients, both specified by investigator as cancer of sigmoid colon. ^3^No information available for 63 patients. ^4^Less than 100 cigarettes in life. ^5^Permanently stopped smoking before colorectal resection surgery (before cancer was suspected or after cancer was suspected but before surgery). ^6^Current smoker at time of colorectal resection surgery (incl. patients who permanently stopped smoking after surgery). ^7^No information available for 6 patients. Additionally, node status is not evaluable for 1 patient; metastasis status is not evaluable for 10 patients. BMI: body mass index, Max: maximum value, Min: minimum value, *N*: total number of patients, *n*: number of patients observed, SD: standard deviation, and TNM classification system: Tumour Node Metastasis classification system.

**Table 2:** Management of intraoperative anastomotic leaks.

Parameter	Total *N* = 458
*Method of diagnosing the intraoperative leak, n (%)*	
Air leak test	347 (75.8%)
Dye test^1^	103 (22.5%)
Others	8 (1.7%)
*Time taken to manage and stop leak (minutes)* ^2^	
*n*	357
Mean (SD)	21.2 (16.8)
*Interventions performed following leak, n (%)* ^3^	
Oversewing of staple line	355 (77.5%)
Sealant	80 (17.5%)
New anastomosis	43 (9.4%)
Ileostomy/colostomy	47 (10.3%)
*Time required for all interventions performed per patient (minutes)* ^4^	
*n*	359
Mean (SD)	22.1 (21.0)

Further details and by-country data provided in Supplemental Table 5. ^1^Methylene blue. ^2^No information available for 101 patients. ^3^Multiple responses possible. No information on interventions available for 14 patients. ^4^No information available for 99 patients. Max: maximum value, Min: minimum value, *N*: total number of patients, *n*: number of patients observed, and SD: standard deviation.
