# Supplementary material for: Diagnosis and Management of Intraoperative Colorectal Anastomotic Leaks: A Global Retrospective Patient Chart Review Study
Source: Surg Res Pract. 2017 Jun 14;2017:3852731. doi: 10.1155/2017/3852731 (PMC5488233; doi:10.1155/2017/3852731)
Supplement: Supplementary file 1 — The supplemental tables provide more granular data, primarily by the each country included in the study. A couple of tables also provide additional details when the data are straitified by the surgical approach, or by the location of the anastomosis. [file 3852731.f1.docx]

# Supporting information

**Supplemental tables:**

[Supplemental Table 1: Patients’ demographic characteristics, by country 2](#_Toc456597018)

[Supplemental Table 2: Patients’ cancer stage before surgery (TNM classification), by country 4](#_Toc456597019)

[Supplemental Table 3: Type of surgical approach, by country 6](#_Toc456597020)

[Supplemental Table 4: Location of anastomosis, by country 7](#_Toc456597021)

[Supplemental Table 5: Management of intra-operative anastomotic leaks, by country 8](#_Toc456597022)

[Supplemental Table 6: Occurrence of post-operative anastomotic leaks, by country 11](#_Toc456597023)

[Supplemental Table 7: Selected results stratified by open or laparoscopic surgery approach 12](#_Toc456597024)

[Supplemental Table 8: Selected results stratified by location of anastomosis in rectum or colon 15](#_Toc456597025)

#### Supplemental Table 1: Patients’ demographic characteristics, by country

| **Characteristic** | **Total** N=458 | **Country** | | | | | | | |
| --- | --- | --- | --- | --- | --- | --- | --- | --- | --- |
|  |  | **USA** N=54 | **Europe** | | | | **Asia** | | |
|  |  |  | **France** N=56 | **Germany** N=62 | **Italy** N=57 | **UK** N=64 | **China** N=57 | **Japan** N=56 | **South Korea** N=52 |
| **Age (years)** |  |  |  |  |  |  |  |  |  |
| n | 458 | 54 | 56 | 62 | 57 | 64 | 57 | 56 | 52 |
| Mean (SD) | 64.51 (9.66) | 63.69 (7.85) | 63.39 (9.63) | 66.63 (8.41) | 62.86 (14.82) | 66.39 (8.93) | 62.14 (9.51) | 65.82 (7.53) | 64.71 (8.04) |
| Median | 65 | 63 | 65 | 67 | 66 | 67 | 64 | 66 | 65 |
| Min, max | 18, 88 | 52, 80 | 45, 82 | 47, 88 | 18, 86 | 46, 84 | 39, 82 | 49, 78 | 50, 87 |
| **Gender, n (% column \ % row)** | | | | | | | | | |
| n | 458 | 54 | 56 | 62 | 57 | 64 | 57 | 56 | 52 |
| Female | 167 (36.5% \ 100%) | 26 (48.1% \ 15.6%) | 27 (48.2% \ 16.2%) | 24 (38.7% \ 14.4%) | 24 (42.1% \ 14.4%) | 21 (32.8% \ 12.6%) | 17 (29.8% \ 10.2%) | 14 (25.0% \ 8.4%) | 14 (26.9% \ 8.4%) |
| Male | 291 (63.5% \ 100%) | 28 (51.9% \ 9.6%) | 29 (51.8% \ 10.0%) | 38 (61.3% \ 13.1%) | 33 (57.9% \ 11.3%) | 43 (67.2% \ 14.8%) | 40 (70.2% \ 13.7%) | 42 (75.0% \ 14.4%) | 38 (73.1% \ 13.1%) |
| **Race, n (% column \ % row)** | | | | | | | | | |
| n | 458 | 54 | 56 | 62 | 57 | 64 | 57 | 56 | 52 |
| White | 259 (56.6% \ 100%) | 35 (64.8% \ 13.5%) | 48 (85.7% \ 18.5%) | 62 (100% \ 23.9%) | 55 (96.5% \ 21.2%) | 59 (92.2% \ 22.8%) | 0 (0.0% \ 0.0%) | 0 (0.0% \ 0.0%) | 0 (0.0% \ 0.0%) |
| Black / African American | 26 (5.7% \ 100%) | 17 (31.5% \ 65.4%) | 5 (8.9% \ 19.2%) | 0 (0.0% \ 0.0%) | 1 (1.8% \ 3.8%) | 3 (4.7% \ 11.5%) | 0 (0.0% \ 0.0%) | 0 (0.0% \ 0.0%) | 0 (0.0% \ 0.0%) |
| Asian | 169 (36.9% \ 100%) | 0 (0.0% \ 0.0%) | 1 (1.8% \ 0.6%) | 0 (0.0% \ 0.0%) | 1 (1.8% \ 0.6%) | 2 (3.1% \ 1.2%) | 57 (100% \ 33.7%) | 56 (100% \ 33.1%) | 52 (100% \ 30.8%) |
| Hispanic | 4 (0.9% \ 100%) | 2 (3.7% \ 50.0%) | 2 (3.6% \ 50.0%) | 0 (0.0% \ 0.0%) | 0 (0.0% \ 0.0%) | 0 (0.0% \ 0.0%) | 0 (0.0% \ 0.0%) | 0 (0.0% \ 0.0%) | 0 (0.0% \ 0.0%) |
| **BMI (kg/m^2^)** |  |  |  |  |  |  |  |  |  |
| n ^1^ | 457 | 53 | 56 | 62 | 57 | 64 | 57 | 56 | 52 |
| Mean (SD) | 25.38 (4.37) | 27.94 (6.11) | 26.57 (3.74) | 25.37 (3.99) | 25.07 (3.57) | 27.91 (4.93) | 24.10 (2.22) | 23.71 (2.38) | 21.96 (3.05) |
| Median | 25 | 27 | 27 | 25 | 25 | 28 | 24 | 24 | 22 |
| Min, max | 17, 48 | 17, 41 | 18, 36 | 19, 38 | 17, 35 | 18, 48 | 18, 29 | 19, 32 | 17, 33 |
| **Type of cancer, n (% column \ % row)** | | | | | | | | | |
| Colon cancer | 300 (65.5% \ 100%) | 47 (87.0% \ 15.7%) | 51 (91.1% \ 17.0%) | 43 (69.4% \ 14.3%) | 31 (54.4% \ 10.3%) | 33 (51.6% \ 11.0%) | 28 (49.1% \ 9.3%) | 42 (75.0% \ 14.0%) | 25 (48.1% \ 8.3%) |
| Rectal cancer | 156 (34.1% \ 100%) | 7 (13.0% \ 4.5%) | 5 (8.9% \ 3.2%) | 17 (27.4% \ 10.9%) | 26 (45.6% \ 16.7%) | 31 (48.4% \ 19.9%) | 29 (50.9% \ 18.6%) | 14 (25.0% \ 9.0%) | 27 (51.9% \ 17.3%) |
| Other ^2^ | 2 (0.4% \ 100%) | 0 (0.0% \ 0.0%) | 0 (0.0% \ 0.0%) | 2 (3.2% \ 100%) | 0 (0.0% \ 0.0%) | 0 (0.0% \ 0.0%) | 0 (0.0% \ 0.0%) | 0 (0.0% \ 0.0%) | 0 (0.0% \ 0.0%) |
| **Smoking status, n (% column \ % row)** ^3^ | | | | | | | | | |
| Never smoked ^4^ | 201 (43.9% \ 100%) | 24 (44.4% \ 11.9%) | 21 (37.5% \ 10.4%) | 21 (33.9% \ 10.4%) | 31 (54.4% \ 15.4%) | 18 (28.1% \ 9.0%) | 26 (45.6% \ 12.9%) | 29 (51.8% \ 14.4%) | 31 (59.6% \ 15.4%) |
| Stopped smoking before surgery ^5^ | 133 (29.0% \ 100%) | 16 (29.6% \ 12.0%) | 20 (35.7% \ 15.0%) | 24 (38.7% \ 18.0%) | 11 (19.3% \ 8.3%) | 12 (18.8% \ 9.0%) | 20 (35.1% \ 15.0%) | 20 (35.7% \ 15.0%) | 10 (19.2% \ 7.5%) |
| Smoking at time of surgery ^6^ | 61 (13.3% \ 100%) | 8 (14.8% \ 13.1%) | 9 (16.1% \ 14.8%) | 9 (14.5% \ 14.8%) | 10 (17.5% \ 16.4%) | 7 (10.9% \ 11.5%) | 10 (17.5% \ 16.4%) | 3 (5.4% \ 4.9%) | 5 (9.6% \ 8.2%) |
| ^1^ BMI: no information available due to missing height measurement for 1 patient (from USA). ^2^ “Other” type documented for 2 patients, both specified by investigator as cancer of sigmoid colon. ^3^ Smoking status: no information available for 63 patients (6 from USA, 6 from France, 8 from Germany, 5 from Italy, 27 from UK, 1 from China, 4 from Japan, 6 from South Korea). ^4^ Less than 100 cigarettes in life. ^5^ Permanently stopped smoking before colorectal resection surgery (before cancer was suspected or after cancer was suspected but before surgery). ^6^ Current smoker at time of colorectal resection surgery (incl. patients who permanently stopped smoking after surgery). Abbreviations: BMI: body mass index, Max: maximum value, Min: minimum value, N: total number of patients, n: number of patients observed, SD: standard deviation, USA: United States of Amerika, UK: United Kingdom. | | | | | | | | | |

#### Supplemental Table 2: Patients’ cancer stage before surgery (TNM classification), by country

| **TNM Stage, n (% column \ % row)** | **Total** N=458 | **Country** | | | | | | | |
| --- | --- | --- | --- | --- | --- | --- | --- | --- | --- |
|  |  | **USA** N=54 | **Europe** | | | | **Asia** | | |
|  |  |  | **France** N=56 | **Germany** N=62 | **Italy** N=57 | **UK** N=64 | **China** N=57 | **Japan** N=56 | **South Korea** N=52 |
| **Tumor status** |  |  |  |  |  |  |  |  |  |
| T0 | 18 (3.9% \ 100%) | 1 (1.9% \ 5.6%) | 16 (28.6% \ 88.9%) | 0 (0.0% \ 0.0%) | 0 (0.0% \ 0.0%) | 0 (0.0% \ 0.0%) | 0 (0.0% \ 0.0%) | 1 (1.8% \ 5.6%) | 0 (0.0% \ 0.0%) |
| T1a | 44 (9.6% \ 100%) | 7 (13.0% \ 15.9%) | 5 (8.9% \ 11.4%) | 7 (11.3% \ 15.9%) | 3 (5.3% \ 6.8%) | 10 (15.6% \ 22.7%) | 1 (1.8% \ 2.3%) | 8 (14.3% \ 18.2%) | 3 (5.8% \ 6.8%) |
| T1b | 34 (7.4% \ 100%) | 5 (9.3% \ 14.7%) | 2 (3.6% \ 5.9%) | 8 (12.9% \ 23.5%) | 4 (7.0% \ 11.8%) | 5 (7.8% \ 14.7%) | 1 (1.8% \ 2.9%) | 8 (14.3% \ 23.5%) | 1 (1.9% \ 2.9%) |
| T2a | 113 (24.7% \ 100%) | 21 (38.9% \ 18.6%) | 8 (14.3% \ 7.1%) | 21 (33.9% \ 18.6%) | 11 (19.3% \ 9.7%) | 26 (40.6% \ 23.0%) | 8 (14.0% \ 7.1%) | 17 (30.4% \ 15.0%) | 1 (1.9% \ 0.9%) |
| T2b | 55 (12.0% \ 100%) | 12 (22.2% \ 21.8%) | 9 (16.1% \ 16.4%) | 4 (6.5% \ 7.3%) | 8 (14.0% \ 14.5%) | 7 (10.9% \ 12.7%) | 8 (14.0% \ 14.5%) | 5 (8.9% \ 9.1%) | 2 (3.8% \ 3.6%) |
| T3 | 145 (31.7% \ 100%) | 8 (14.8% \ 5.5%) | 15 (26.8% \ 10.3%) | 18 (29.0% \ 12.4%) | 24 (42.1% \ 16.6%) | 13 (20.3% \ 9.0%) | 22 (38.6% \ 15.2%) | 14 (25.0% \ 9.7%) | 31 (59.6% \ 21.4%) |
| T4 | 43 (9.4% \ 100%) | 0 (0.0% \ 0.0%) | 0 (0.0% \ 0.0%) | 3 (4.8% \ 7.0%) | 6 (10.5% \ 14.0%) | 1 (1.6% \ 2.3%) | 17 (29.8% \ 39.5%) | 2 (3.6% \ 4.7%) | 14 (26.9% \ 32.6%) |
| **Node status** ^1^ | | | | | | | | | |
| N0 | 202 (44.1% \ 100%) | 25 (46.3% \ 12.4%) | 35 (62.5% \ 17.3%) | 19 (30.6% \ 9.4%) | 22 (38.6% \ 10.9%) | 35 (54.7% \ 17.3%) | 25 (43.9% \ 12.4%) | 22 (39.3% \ 10.9%) | 19 (36.5% \ 9.4%) |
| N1 | 172 (37.6% \ 100%) | 21 (38.9% \ 12.2%) | 16 (28.6% \ 9.3%) | 28 (45.2% \ 16.3%) | 23 (40.4% \ 13.4%) | 19 (29.7% \ 11.0%) | 23 (40.4% \ 13.4%) | 28 (50.0% \ 16.3%) | 14 (26.9% \ 8.1%) |
| N2 | 66 (14.4% \ 100%) | 4 (7.4% \ 6.1%) | 4 (7.1% \ 6.1%) | 10 (16.1% \ 15.2%) | 10 (17.5% \ 15.2%) | 7 (10.9% \ 10.6%) | 7 (12.3% \ 10.6%) | 5 (8.9% \ 7.6%) | 19 (36.5% \ 28.8%) |
| N3 | 11 (2.4% \ 100%) | 4 (7.4% \ 36.4%) | 0 (0.0% \ 0.0%) | 4 (6.5% \ 36.4%) | 1 (1.8% \ 9.1%) | 1 (1.6% \ 9.1%) | 1 (1.8% \ 9.1%) | 0 (0.0% \ 0.0%) | 0 (0.0% \ 0.0%) |
| **Metastasis status** ^2^ | | | | | | | | | |
| M0 | 411 (89.7% \ 100%) | 45 (83.3% \ 10.9%) | 55 (98.2% \ 13.4%) | 41 (66.1% \ 10.0%) | 50 (87.7% \ 12.2%) | 61 (95.3% \ 14.8%) | 57 (100% \ 13.9%) | 53 (94.6% \ 12.9%) | 49 (94.2% \ 11.9%) |
| M1 | 31 (6.8% \ 100%) | 5 (9.3% \ 16.1%) | 1 (1.8% \ 3.2%) | 14 (22.6% \ 45.2%) | 6 (10.5% \ 19.4% | 1 (1.6% \ 3.2%) | 0 (0.0% \ 0.0%) | 1 (1.8% \ 3.2%) | 3 (5.8% \ 9.7%) |
| No details regarding TNM status were available for 6 patients (1 each from Germany, France, Japan, and Italy; 2 from the UK). ^1^ Node status not evaluable for 1 patient (from China). ^2^ Metastasis status not evaluable for 10 patients (4 from USA, 6 from Germany). Abbreviations: N: total number of patients, n: number of patients observed, TNM: Tumour Node Metastasis classification system, USA: United States of Amerika, UK: United Kingdom. | | | | | | | | | |

#### Supplemental Table 3: Type of surgical approach, by country

| **Country** | **N** | **Type of surgical approach** | | | | |
| --- | --- | --- | --- | --- | --- | --- |
|  |  | **Open** n (%) | **Laparoscopic** n (%) | **Hand-assisted** n (%) | **Robotic** n (%) | **Converted** n (%) |
| **Total** | 458 | 164 (35.8%) | 264 (57.6%) | 28 (6.1%) | 9 (2.0%) | 9 (2.0%) |
| **USA** | 54 | 18 (33.3%) | 28 (51.9%) | 5 (9.3%) | 1 (1.9%) | 2 (3.7%) |
| **Europe** |  |  |  |  |  |  |
| France | 56 | 8 (14.3%) | 44 (78.6%) | 2 (3.6%) | 0 (0.0%) | 3 (5.4%) |
| Germany | 62 | 18 (29.0%) | 39 (62.9%) | 0 (0.0%) | 4 (6.5%) | 2 (3.2%) |
| Italy | 57 | 16 (28.1%) | 37 (64.9%) | 2 (3.5%) | 4 (7.0%) | 2 (3.5%) |
| UK | 64 | 32 (50.0%) | 27 (42.2%) | 5 (7.8%) | 0 (0.0%) | 0 (0.0%) |
| **Asia** |  |  |  |  |  |  |
| China | 57 | 26 (45.6%) | 29 (50.9%) | 2 (3.5%) | 0 (0.0%) | 0 (0.0%) |
| Japan | 56 | 19 (33.9%) | 35 (62.5%) | 2 (3.6%) | 0 (0.0%) | 0 (0.0%) |
| South Korea | 52 | 27 (51.9%) | 25 (48.1%) | 10 (19.2%) | 0 (0.0%) | 0 (0.0%) |
| Abbreviations: N: total number of patients, n: number of patients observed, USA: United States of Amerika, UK: United Kingdom. | | | | | | |

#### Supplemental Table 4: Location of anastomosis, by country

| **Country, n (% row \ % column)** | **N** | **Location of anastomosis** ^1^ | | | |
| --- | --- | --- | --- | --- | --- |
|  |  | **Rectum n (%)** | **Descending colon n (%)** | **Transverse colon n (%)** | **Ascending colon n (%)** |
| **Total** | 458 | 262 (57.2% \ 100%) | 130 (28.4% \ 100%) | 45 (9.8% \ 100%) | 19 (4.1% \ 100%) |
| **USA** | 54 | 12 (22.2% \ 4.6%) | 22 (40.7% \ 16.9%) | 12 (22.2% \ 26.7%) | 7 (13.0% \ 36.8%) |
| **Europe** |  |  |  |  |  |
| France | 56 | 29 (51.8% \ 11.1%) | 21 (37.5% \ 16.2%) | 6 (10.7% \ 13.3%) | 0 (0.0% \ 0.0%) |
| Germany | 62 | 31 (50.0% \ 11.8%) | 13 (21.0% \ 10.0%) | 13 (21.0% \ 28.9%) | 5 (8.1% \ 26.3%) |
| Italy | 57 | 31 (54.4% \ 11.8%) | 17 (29.8% \ 13.1%) | 4 (7.0% \ 8.9%) | 4 (7.0% \ 21.1%) |
| UK | 64 | 45 (70.3% \ 17.2%) | 14 (21.9% \ 10.8%) | 4 (6.3% \ 8.9%) | 1 (1.6% \ 5.3%) |
| **Asia** |  |  |  |  |  |
| China | 57 | 31 (54.4% \ 11.8%) | 21 (36.8% \ 16.2%) | 5 (8.8% \ 11.1%) | 0 (0.0% \ 0.0%) |
| Japan | 56 | 39 (69.6% \ 14.9%) | 15 (26.8% \ 11.5%) | 0 (0.0% \ 0.0%) | 2 (3.6% \ 10.5%) |
| South Korea | 52 | 44 (84.6% \ 16.8%) | 7 (13.5% \ 5.4%) | 1 (1.9% \ 2.2%) | 0 (0.0% \ 0.0%) |
| ^1^ “Other” location documented for 2 patients (1 from USA, 1 from Italy; both specified by investigator as sigmoid colon).  Abbreviations: N: total number of patients, n: number of patients observed, USA: United States of Amerika, UK: United Kingdom. | | | | | |

#### Supplemental Table 5: Management of intra-operative anastomotic leaks, by country

| **Parameter** | **Total** N=458 | **Country** | | | | | | | |
| --- | --- | --- | --- | --- | --- | --- | --- | --- | --- |
|  |  | **USA** N=54 | **Europe** | | | | **Asia** | | |
|  |  |  | **France** N=56 | **Germany** N=62 | **Italy** N=57 | **UK** N=64 | **China** N=57 | **Japan** N=56 | **S. Korea** N=52 |
| **Method of diagnosing the intra-operative leak, n (% column \ % row)** | | | | | | | | | |
| Air leak test | 347 (75.8% \ 100%) | 41 (75.9% \ 11.8%) | 27 (48.2% \ 7.8%) | 43 (69.4% \ 12.4%) | 31 (54.4% \ 8.9%) | 64 (100% \ 18.4%) | 45 (78.9% \ 13.0%) | 48 (85.7% \ 13.8%) | 48 (92.3% \ 13.8%) |
| Dye test ^1^ | 103 (22.5% \ 100%) | 12 (22.2% \ 11.7%) | 26 (46.4% \ 25.2%) | 17 (27.4% \ 16.5%) | 26 (45.6% \ 25.2%) | 0 (0.0% \ 0.0%) | 12 (21.1% \ 11.7%) | 8 (14.3% \ 7.8%) | 2 (3.8% \ 1.9%) |
| Other | 8 (1.7% \ 100%) | 1 (1.9% \ 12.5%) | 3 (5.4% \ 37.5%) | 2 (3.2% \ 25.0%) | 0 (0.0% \ 0.0%) | 0 (0.0% \ 0.0%) | 0 (0.0% \ 0.0%) | 0 (0.0% \ 0.0%) | 2 (3.8% \ 25.0%) |
| **Time taken to manage and stop leak (minutes)** ^2^ | | | | | | | | | |
| n | 357 | 24 | 54 | 50 | 53 | 30 | 54 | 50 | 42 |
| Mean (SD) | 21.22 (16.75) | 19.00 (11.40) | 13.22 (13.05) | 21.60 (13.70) | 24.58 (13.39) | 23.40 (22.57) | 27.70 (20.45) | 12.28 (8.20) | 28.86 (19.80) |
| Median | 17 | 15 | 10 | 20 | 20 | 15 | 25 | 11 | 30 |
| Min, max | 1, 120 | 4, 40 | 1, 60 | 2, 60 | 3, 60 | 5, 120 | 2, 80 | 1, 30 | 1, 120 |
| **Interventions performed following leak, n (% column) ^3^** | | | | | | | | | |
| Oversewing of staple line | 355 (77.5%) | 48 (88.9%) | 32 (57.1%) | 33 (53.2%) | 42 (73.7%) | 51 (79.7%) | 54 (94.7%) | 53 (94.6%) | 42 (80.8%) |
| Sealant | 80 (17.5%) | 5 (9.3%) | 19 (33.9%) | 15 (24.2%) | 19 (33.3%) | 2 (3.1%) | 4 (7.0%) | 6 (10.7%) | 10 (19.2%) |
| New anastomosis | 43 (9.4%) | 3 (5.6%) | 3 (5.4%) | 15 (24.2%) | 8 (14.0%) | 4 (6.3%) | 0 (0.0%) | 6 (10.7%) | 4 (7.7%) |
| Ileostomy / colostomy | 47 (10.3%) | 0 (0.0%) | 6 (10.7%) | 6 (9.7%) | 11 (19.3%) | 12 (18.8%) | 5 (8.8%) | 1 (1.8%) | 6 (11.5%) |
| **Number of suture strands used for oversewing ^4^** | | | | | | | | | |
| n | 282 | 12 | 31 | 32 | 38 | 29 | 52 | 51 | 37 |
| Mean (SD) | 3.9 (3.2) | 4.7 (2.2) | 2.6 (1.7) | **2.9 (1.5)** | 3.5 (3.3) | 2.7 (2.2) | 4.8 (3.7) | 3.7 (2.1) | 5.9 (5.1) |
| Median | 3 | 4 | 2 | 3 | 3 | 2 | 4 | 3 | 5 |
| Min, max | 1, 26 | 1, 10 | 1, 10 | 1, 6 | 1, 20 | 1, 12 | 1, 15 | 1, 12 | 1, 26 |
| **Time required for oversewing, per patient (minutes) ^4^** | | | | | | | | | |
| n | 282 | 12 | 31 | 32 | 38 | 29 | 52 | 51 | 37 |
| Mean (SD) | 15.8 (10.2) | 16.3 (8.4) | 18.7 (14.6) | 17.1 (12.5) | 15.7 (8.0) | 11.6 (7.4) | 19.6 (11.4) | 11.4 (6.9) | 16.4 (6.8) |
| Median | 15 | 15 | 15 | 15 | 15 | 10 | 20 | 10 | 15 |
| Min, max | 1, 75 | 3, 30 | 5, 75 | 2, 50 | 2, 30 | 5, 30 | 2, 60 | 1, 30 | 5, 30 |
| **Number of sealant tubes used ^5^** | | | | | | | | | |
| n | 67 | 3 | 19 | 14 | 17 | 2 | 3 | 6 | 3 |
| Mean (SD) | 2.2 (1.0) | 2.0 (0.0) | 2.4 (0.8) | 2.2 (1.1) | 2.1 (1.2) | 1.5 (0.7) | 3.0 (1.0) | 1.8 (0.8) | 1.3 (0.6) |
| Median | 2 | 2 | 2 | 2 | 2 | 2 | 3 | 2 | 1 |
| Min, max | 1, 6 | 2, 2 | 1, 4 | 1, 5 | 1, 6 | 1, 2 | 2, 4 | 1, 3 | 1, 2 |
| **Time required for intervention using sealant, per patient (minutes) ^5^** | | | | | | | | | |
| n | 67 | 3 | 19 | 14 | 17 | 2 | 3 | 6 | 3 |
| Mean (SD) | 14.9 (13.1) | 26.0 (9.6) | 4.3 (1.3) | 30.7 (14.0) | 13.5 (8.6) | 13.5 (2.1) | 16.7 (12.6) | 6.7 (3.7) | 21.7 (14.4) |
| Median | 10 | 30 | 5 | 40 | 12 | 14 | 15 | 6 | 30 |
| Min, max | 2, 50 | 15, 33 | 2, 6 | 5, 50 | 3, 40 | 12, 15 | 5, 30 | 3, 12 | 5, 30 |
| **Device used for creation of a new anastomosis, n (%) ^6^** | | | | | | | | | |
| Circular stapler | 33 (76.7%) | 3 (100.0%) | 3 (100.0%) | 11 (73.3%) | 6 (75.0%) | 4 (100.0%) | 0 (0.0%) | 3 (50.0%) | 3 (75.0%) |
| Linear stapler | 7 (16.3%) | 0 (0.0%) | 2 (66.7%) | 1 (6.7%) | 2 (25.0%) | 1 (25.0%) | 0 (0.0%) | 0 (0.0%) | 1 (25.0%) |
| Sutures | 9 (20.9%) | 0 (0.0%) | 0 (0.0%) | 4 (26.7%) | 0 (0.0%) | 0 (0.0%) | 0 (0.0%) | 3 (50.0%) | 2 (50.0%) |
| Other | 2 (4.7%) | 0 (0.0%) | 0 (0.0%) | 0 (0.0%) | 0 (0.0%) | 0 (0.0%) | 0 (0.0%) | 0 (0.0%) | 2 (50.0%) |
| **Time required for creation of new anastomosis, per patient (minutes) ^7^** | | | | | | | | | |
| n | 37 | 3 | 3 | 12 | 7 | 2 | 0 | 6 | 4 |
| Mean (SD) | 37.0 (21.8) | 35.0 (5.0) | 60.0 (0.0) | 35.4 (20.1) | 34.6 (17.0) | 75.0 (63.6) | - | 25.8 (15.3) | 27.5 (5.0) |
| Median | 30 | 35 | 60 | 30 | 30 | 75 | - | 20 | 30 |
| Min, max | 10, 120 | 30, 40 | 60, 60 | 10, 70 | 12, 60 | 30, 120 | - | 15, 55 | 20, 30 |
| **Time required for ileostomy / colostomy, per patient (minutes) ^8^** | | | | | | | | | |
| n | 37 | 0 | 5 | 5 | 11 | 9 | 4 | 1 | 2 |
| Mean (SD) | 29.4 (23.6) | - | 22.8 (6.8) | 26.0 (5.5) | 36.8 (42.4) | 27.2 (9.1) | 26.3 (4.8) | 30.0 (-) | 30.0 (0.0) |
| Median | 30 | - | 20 | 30 | 25 | 30 | 28 | 30 | 30 |
| Min, max | 10, 160 | - | 15, 30 | 20, 30 | 10, 160 | 10, 40 | 20, 30 | 30, 30 | 30, 30 |
| **Time required for all interventions performed per patient (minutes)** ^9^ | | | | | | | | | |
| n | 359 | 17 | 48 | 58 | 53 | 36 | 54 | 54 | 39 |
| Mean (SD) | 22.05 (21.04) | 22.24 (11.82) | 19.92 (19.87) | 26.43 (18.50) | 27.77 (36.05) | 21.06 (22.75) | 21.70 (14.92) | 14.93 (11.27) | 21.59 (14.66) |
| Median | 17 | 25 | 10 | 20 | 20 | 14 | 20 | 14 | 20 |
| Min, max | 1, 265 | 3, 40 | 2, 105 | 2, 80 | 2, 265 | 2, 120 | 2, 75 | 1, 55 | 5, 80 |
| ^1^ Methylene blue. ^2^ No information available for 101 patients (30 from USA, 2 from France, 12 from Germany, 4 from Italy, 34 from UK, 3 from China, 6 from Japan, 10 from South Korea). ^3^ Multiple responses possible. No information on interventions available for 14 patients (7 from France, 1 from UK, 6 from South Korea). ^4^ No information available for 73 patients (36 from USA, 1 from France, 1 from Germany, 4 from Italy, 22 from UK, 2 from China, 2 from Japan, 5 from South Korea). ^5^ No information available for 13 patients (2 from USA, 1 from Germany, 2 from Italy, 1 from China, 7 from South Korea). ^6^ Multiple responses possible. ^7^ No information available for 6 patients (3 from Germany, 1 from Italy, 2 from UK). ^8^ No information available for 10 patients (1 from France, 1 from Germany, 3 from UK, 1 from China, 4 from South Korea). ^9^ No information available for 99 patients (37 from USA, 8 from France, 4 from Germany, 4 from Italy, 28 from UK, 3 from China, 2 from Japan, 13 from South Korea). Abbreviations: Max: maximum value, Min: minimum value, N: total number of patients, n: number of patients observed, SD: standard deviation, USA: United States of Amerika, UK: United Kingdom. | | | | | | | | | |

#### Supplemental Table 6: Occurrence of post-operative anastomotic leaks, by country

| **Country** | **N** n (% row \ % column) | **Occurrence of a post-operative anastomotic leak?** | |
| --- | --- | --- | --- |
|  |  | **Yes** n (% row \ % column) | **No** n (% row \ % column) |
| **Total** | 458 (100% \ 100%) | 62 (13.5% \ 100%) | 396 (86.5% \ 100%) |
| **USA** | 54 (100% \ 11.8%) | 6 (11.1% \ 9.7%) | 48 (88.9% \ 12.1%) |
| **Europe** |  |  |  |
| France | 56 (100% \ 12.2%) | 2 (3.6% \ 3.2%) | 54 (96.4% \ 13.6%) |
| Germany | 62 (100% \13.5%) | 7 (11.3% \ 11.3%) | 55 (88.7% \ 13.9%) |
| Italy | 57 (100% \ 12.4%) | 14 (24.6% \ 22.6%) | 43 (75.4% \ 10.9%) |
| UK | 64 (100% \ 14.0%) | 8 (12.5% \ 12.9%) | 56 (87.5% \ 14.1%) |
| **Asia** |  |  |  |
| China | 57 (100% \ 12.4%) | 10 (17.5% \ 16.1%) | 47 (82.5% \ 11.9%) |
| Japan | 56 (100% \ 12.2%) | 8 (14.3% \ 12.9%) | 48 (85.7% \ 12.1%) |
| South Korea | 52 (100% \ 11.4%) | 7 (13.5% \ 11.3%) | 45 (86.5% \ 11.4%) |
| Abbreviations: N: total number of patients, n: number of patients observed, USA: United States of Amerika, UK: United Kingdom. | | | |

#### **Supplemental Table 7:** Selected results stratified by open or laparoscopic surgery approach

| **Characteristic** | **Total** ^1^ N=428 | **Open surgery** N=164 | **Laparoscopic surgery** N=264 |
| --- | --- | --- | --- |
| **Age (years)** |  |  |  |
| Mean (SD) | 64.46 (9.40) | 66.02 (9.59) | 63.48 (9.17) |
| Median | 65 | 67 | 65 |
| Min, max | 27, 88 | 27, 88 | 35, 86 |
| **BMI (kg/m^2^)** |  |  |  |
| n ^2^ | 427 | 163 | 264 |
| Mean (SD) | 25.15 (4.16) | 24.71 (3.89) | 25.42 (4.31) |
| Median | 25 | 24 | 25 |
| Min, max | 17, 48 | 18, 39 | 17, 48 |
| **Country, n (% row \ % column)** *(p-value: 0.0004)* |  |  |  |
| **USA** | 46 (100% \ 10.7%) | 18 (39.1% \ 11.0%) | 28 (60.9% \ 10.6%) |
| **Europe** |  |  |  |
| France | 52 (100% \ 12.1%) | 8 (15.4% \ 4.9%) | 44 (84.6% \ 16.7%) |
| Germany | 57 (100% \ 13.3%) | 18 (31.6% \ 11.0%) | 39 (68.4% \ 14.8%) |
| Italy | 53 (100% \ 12.4%) | 16 (30.2% \ 9.8%) | 37 (69.8% \ 14.0%) |
| UK | 59 (100% \ 13.8%) | 32 (54.2% \ 19.5%) | 27 (45.8% \ 10.2%) |
| **Asia** |  |  |  |
| China | 55 (100% \ 12.9%) | 26 (47.3% \ 15.9%) | 29 (52.7% \ 11.0%) |
| Japan | 54 (100% \ 12.6%) | 19 (35.2% \ 11.6%) | 35 (64.8% \ 13.3%) |
| South Korea | 52 (100% \ 12.1%) | 27 (51.9% \ 16.5%) | 25 (48.1% \ 9.5%) |
| **Type of cancer, n (% column \ % row)** *(p-value: <0.0001)* |  |  |  |
| Colon cancer | 282 (65.9% \ 100%) | 88 (53.7% \ 31.2%) | 194 (73.5% \ 68.8%) |
| Rectal cancer | 144 (33.6% \ 100%) | 76 (46.3% \ 52.8%) | 68 (25.8% \ 47.2%) |
| Other ^3^ | 2 (0.5% \ 100%) | 0 (0.0% \ 0.0%) | 2 (0.8% \ 100%) |
| **Location of anastomosis, n (% column \ % row)** *(p-value: 0.0159)* |  |  |  |
| Ascending colon | 17 (4.0% \ 100%) | 3 (1.8% \ 17.6%) | 14 (5.3% \ 82.4%) |
| Transverse colon | 44 (10.3% \ 100%) | 10 (6.1% \ 22.7%) | 34 (12.9% \ 77.3%) |
| Descending colon | 121 (28.3% \ 100%) | 44 (26.8% \ 36.4%) | 77 (29.2% \ 63.6%) |
| Rectum | 244 (57.0% \ 100%) | 107 (65.2% \ 43.9%) | 137 (51.9% \ 56.1%) |
| Other ^3^ | 2 (0.5% \ 100%) | 0 (0.0% \ 0.0%) | 2 (0.8% \ 100%) |
| **Time taken to manage and stop intra-operative anastomotic leak (minutes)** |  |  |  |
| n | 335 | 117 | 218 |
| Mean (SD) | 21.19 (16.97) | 22.30 (16.99) | 20.59 (16.97) |
| Median | 15 | 20 | 15 |
| Min, max | 1, 120 | 1, 80 | 1, 120 |
| **Interventions performed following intra-operative leak, n (%)** |  |  |  |
| n | 414 | 164 | 250 |
| Oversewing of staple line | 333 (77.80%) | 143 (87.20%) | 190 (71.97%) |
| Sealant | 76 (17.76%) | 12 (7.32%) | 64 (24.24%) |
| New anastomosis | 35 (8.18%) | 17 (10.37%) | 18 (6.82%) |
| Ileostomy / colostomy | 43 (10.05%) | 16 (9.76%) | 27 (10.23%) |
| **Duration of entire surgery** |  |  |  |
| n | 427 | 164 | 263 |
| Mean (SD) | 190.38 (83.46) | 184.96 (65.66) | 193.77 (92.81) |
| Median | 180 | 180 | 180 |
| Min, max | 45, 1098 | 45, 446 | 47, 1098 |
| **Duration of hospitalisation (days)** |  |  |  |
| n | 413 | 155 | 258 |
| Mean (SD) | 20.39 (30.31) | 22.95 (41.93) | 18.85 (20.33) |
| Median | 14 | 15 | 13 |
| Min, max | 1, 393 | 1, 393 | 2, 171 |
| **Duration of from hospital admission to surgery (days)** |  |  |  |
| n | 428 | 164 | 264 |
| Mean (SD) | 4.98 (13.52) | 3.97 (8.93) | 5.61 (15.70) |
| Median | 2 | 2 | 2 |
| Min, max | 0, 158 | 0, 96 | 0, 158 |
| **Duration from surgery to hospital discharge (days)** |  |  |  |
| n | 413 | 155 | 258 |
| Mean (SD) | 15.51 (27.41) | 19.19 (41.19) | 13.31 (13.22) |
| Median | 11 | 12 | 10 |
| Min, max | 0, 393 | 0, 393 | 0, 150 |
| **Patient survival, n (% column \ % row)** *(p-value: 0.0043)* |  |  |  |
| Patient survived | 423 (98.8% \ 100%) | 159 (97.0% \ 37.6%) | 264 (100% \ 62.4%) |
| Patient died | 5 (1.2% \ 100%) | 5 (3.0% \ 100%) | 0 (0.0% \ 0.0%) |
| **Occurrence of post-operative anastomotic leak,  n (% column \ % row)** *(p-value: 0.0019)* |  |  |  |
| Yes | 56 (13.1% \ 100%) | 32 (19.5% \ 57.1%) | 24 (9.1% \ 42.9%) |
| No | 372 (86.9% \ 100%) | 132 (80.5% \ 35.5%) | 240 (90.9% \ 64.5%) |
| P-values (Chi square test) shown if available / calculated.  ^1^ Total number of patients is 428, as for 30 patients (6.6%) overall neither laparoscopic nor open approach was documented.  ^2^ No information available due to missing height measurement for 1 patient (from USA) in open surgery group.  ^3^ “Other” cancer / location specified by investigator as cancer of sigmoid colon in both cases.  Abbreviations: BMI: body mass index, Max: maximum value, Min: minimum value, N: total number of patients, n: number of patients observed, SD: standard deviation, USA: United States of Amerika, UK: United Kingdom. | | | |

#### Supplemental Table 8: Selected results stratified by location of anastomosis in rectum or colon

| **Characteristic** | **Total** N=458 | **Location of anastomosis in rectum** N=262 | **Location of anastomosis in colon** N=196 |
| --- | --- | --- | --- |
| **Age (years)** |  |  |  |
| Mean (SD) | 64.51 (9.66) | 65.60 (9.69) | 63.06 (9.45) |
| Median | 65 | 66 | 64 |
| Min, max | 18, 88 | 18, 88 | 27, 84 |
| **BMI (kg/m^2^)** |  |  |  |
| n ^1^ | 457 | 262 | 195 |
| Mean (SD) | 25.38 (4.37) | 25.33 (4.26) | 25.46 (4.52) |
| Median | 25 | 25 | 25 |
| Min, max | 17, 48 | 18, 48 | 17, 42 |
| **Country, n (% row \ % column)** *(p-value: <0.0001)* |  |  |  |
| **USA** | 54 (100% \ 11.8%) | 12 (22.2% \ 4.6%) | 42 (77.8% \ 21.4%) |
| **Europe** |  |  |  |
| France | 56 (100% \ 12.2%) | 29 (51.8% \ 11.1%) | 27 (48.2% \ 13.8%) |
| Germany | 62 (100% \ 13.5%) | 31 (50.0% \ 11.8%) | 31 (50.0% \ 15.8%) |
| Italy | 57 (100% \ 12.4%) | 31 (54.4% \ 11.8%) | 26 (45.6% \ 13.3%) |
| UK | 64 (100% \ 14.0%) | 45 (70.3% \ 17.2%) | 19 (29.7% \ 9.7%) |
| **Asia** |  |  |  |
| China | 57 (100% \ 12.4%) | 31 (54.4% \ 11.8%) | 26 (45.6% \ 13.3%) |
| Japan | 56 (100% \ 12.2%) | 39 (69.6% \ 14.9%) | 17 (30.4% \ 8.7%) |
| South Korea | 52 (100% \ 11.4%) | 44 (84.6% \ 16.8%) | 8 (15.4% \ 4.1%) |
| **Type of cancer, n (% column \ % row)** *(p-value: <0.0001)* |  |  |  |
| Colon cancer | 300 (65.5% \ 100%) | 126 (48.1% \ 42.0%) | 174 (88.8% \ 58.0%) |
| Rectal cancer | 156 (34.1% \ 100%) | 135 (51.5% \ 86.5%) | 21 (10.7% \ 13.5%) |
| Other ^2^ | 2 (0.4% \ 100%) | 1 (0.4% \ 50.0%) | 1 (0.5% \ 50.0%) |
| **Surgical approach, n (%)** |  |  |  |
| Open | 164 (35.81%) | 107 (40.84%) | 57 (29.08%) |
| Laparoscopic | 264 (57.64%) | 137 (52.29%) | 127 (64.80%) |
| Hand-assisted | 28 (6.11%) | 18 (6.87%) | 10 (5.10%) |
| Robotic | 9 (1.97%) | 7 (2.67%) | 2 (1.02%) |
| Converted | 9 (1.97%) | 7 (2.67%) | 2 (1.02%) |
| **Time taken to manage and stop intra-operative anastomotic leak (minutes)** |  |  |  |
| n | 357 | 212 | 145 |
| Mean (SD) | 21.22 (16.75) | 21.92 (17.71) | 20.21 (15.23) |
| Median | 17 | 15 | 18 |
| Min, max | 1, 120 | 1, 120 | 1, 70 |
| **Interventions performed following intra-operative leak, n (%)** |  |  |  |
| n | 458 | 262 | 196 |
| Oversewing of staple line | 355 (77.51%) | 205 (78.24%) | 150 (76.53%) |
| Sealant | 80 (17.47%) | 34 (12.98%) | 46 (23.47%) |
| New anastomosis | 43 (9.39%) | 25 (9.54%) | 18 (9.18%) |
| Ileostomy / colostomy | 47 (10.26%) | 42 (16.03%) | 5 (2.55%) |
| **Duration of entire surgery** |  |  |  |
| n | 457 | 262 | 195 |
| Mean (SD) | 190.05 (83.13) | 199.36 (74.28) | 177.55 (92.45) |
| Median | 180 | 187 | 170 |
| Min, max | 45, 1098 | 62, 480 | 45, 1098 |
| **Duration of hospitalisation (days)** |  |  |  |
| n | 443 | 252 | 191 |
| Mean (SD) | 19.94 (29.37) | 22.30 (31.53) | 16.83 (26.00) |
| Median | 14 | 15 | 12 |
| Min, max | 1, 393 | 4, 393 | 1, 339 |
| **Duration of from hospital admission to surgery (days)** |  |  |  |
| n | 458 | 262 | 196 |
| Mean (SD) | 4.85 (13.12) | 5.45 (15.81) | 4.05 (8.24) |
| Median | 2 | 2 | 2 |
| Min, max | 0, 158 | 0, 158 | 0, 63 |
| **Duration from surgery to hospital discharge (days)** |  |  |  |
| n | 443 | 252 | 191 |
| Mean (SD) | 15.19 (26.53) | 17.00 (27.60) | 12.81 (24.93) |
| Median | 10 | 12 | 9 |
| Min, max | 0, 393 | 0, 393 | 0, 338 |
| **Patient survival, n (% column \ % row)** *(p-value: 0.3003)* |  |  |  |
| Patient survived | 453 (98.9% \ 100%) | 258 (98.5% \ 57.0%) | 195 (99.5% \ 43.0%) |
| Patient died | 5 (1.1% \ 100%) | 4 (1.5% \ 80.0%) | 1 (0.5% \ 20.0%) |
| **Occurrence of post-operative anastomotic leak,  n (% column \ % row)** *(p-value: 0.0002)* |  |  |  |
| Yes | 62 (13.5% \ 100%) | 49 (18.7% \ 79.0%) | 13 (6.6% \ 21.0%) |
| No | 396 (86.5% \ 100%) | 213 (81.3% \ 53.8%) | 183 (93.4% \ 46.2%) |
| P-values (Chi square test) shown if available / calculated.  ^1^ No information available due to missing height measurement for 1 patient (from USA) among patients with anastomosis in colon.  ^2^ “Other” cancer / location specified by investigator as cancer of sigmoid colon in both cases.  Abbreviations: BMI: body mass index, Max: maximum value, Min: minimum value, N: total number of patients, n: number of patients observed, SD: standard deviation, USA: United States of Amerika, UK: United Kingdom. | | | |
